# Motivating Factors and Barriers Toward Choosing a Career in Ophthalmology: A Narrative Review of the Literature

**DOI:** 10.7759/cureus.112811

**Published:** 2026-07-16

**Authors:** Wasse Uddin Ahmed Saleh

**Affiliations:** 1 General Surgery, Nottingham University NHS Trust, Nottingham, GBR

**Keywords:** barriers, career choice, medical students, motivating factors, ophthalmology, specialty selection, workforce

## Abstract

Ophthalmology combines microsurgical precision with rapidly advancing technology, offering profound patient impact through vision-restoring interventions. Despite these compelling qualities, there are growing global concerns about the sustainability of the ophthalmic workforce in the face of rising demand for eye care. This narrative review synthesizes published evidence on the motivating factors and barriers influencing medical students and junior doctors in choosing a career in ophthalmology, drawing on studies examining both surgical career choice broadly and ophthalmology-specific literature. Key motivating factors identified include the intellectual challenge of the specialty, exposure to innovative surgical procedures, the influence of positive role models and mentors, rewarding patient outcomes, and a comparatively favorable lifestyle profile within surgery. Significant barriers include limited undergraduate exposure to ophthalmology, the perceived competitiveness of training pathways, financial burdens of training, gender and diversity inequities, and insufficient structured mentorship. Addressing these barriers through early targeted exposure, inclusive mentorship, and curriculum reform is essential to securing a sustainable ophthalmic workforce. Ophthalmology societies, medical schools, and training bodies must collaborate to communicate the specialty’s unique rewards and to dismantle structural inequities in access to training.

PubMed/MEDLINE, Embase, and Google Scholar were searched from January 1995 to March 2025, supplemented by manual screening of reference lists and relevant gray literature (professional body reports and workforce surveys). Search terms used were "ophthalmology", "surgery", "career choice", "specialty selection", "medical students", "mentorship", and "workforce." Ophthalmology-specific studies were prioritized where available; broader surgical career-choice literature, most notably the general surgery framework of Hossain and Hossain, was drawn upon selectively to address areas where ophthalmology-specific evidence was sparse. As a narrative rather than systematic review, study selection was not exhaustive and reflects the authors' judgment of relevance and quality rather than a predefined, reproducible screening protocol.

## Introduction and background

Ophthalmology occupies a singular position within medicine, seamlessly integrating the precision of microsurgery with cutting-edge diagnostic technology and pharmacotherapeutic innovation. From phacoemulsification cataract surgery -- widely recognized as among the most cost-effective health interventions in the world -- to gene therapy for inherited retinal dystrophies and artificial intelligence-based screening for diabetic eye disease, the specialty demands both exceptional manual dexterity and rigorous scientific literacy. Yet despite these compelling attributes, there is growing international concern that the pipeline of newly trained ophthalmologists may prove insufficient to meet the mounting global burden of visual impairment projected over the coming decades.

The determinants of specialty choice in medicine are well established as multifactorial. A landmark survey of over 2,000 Canadian medical students identified five principal domains influencing career decisions: medical lifestyle, social orientation, prestige, hospital orientation, and the availability of role models and a varied scope of practice [1]. These findings resonate across international settings. In the United States, a survey across 16 medical schools identified lifestyle and workload as the principal negative influences on students contemplating a surgical career [2]. A survey of over 2,000 Canadian medical students further identified the role of mentors, career opportunities, and scope of practice as central motivators [3].

General surgery has experienced a documented global decline in trainee interest, attributed to lifestyle concerns, reduced undergraduate exposure, and shifting workforce demographics [4,5]. Whether parallel forces apply specifically to ophthalmology has received comparatively limited systematic attention in the literature [6]. Ophthalmology faces a structural disadvantage: clinical placements are frequently brief or absent from undergraduate medical curricula in many countries, meaning that students may graduate with little substantive exposure to the specialty before making career decisions.

This article reviews the published evidence on the factors that motivate medical students to consider ophthalmology as a career, and the barriers that deter them from doing so. It draws on the broader surgical career choice literature -- most notably the framework articulated by Hossain and Hossain [4] -- and applies these themes to the specific context of ophthalmology, with the aim of informing evidence-based strategies for recruitment and workforce planning.

Given the narrative rather than systematic nature of this review, inclusion and exclusion criteria were applied pragmatically rather than through a predefined PRISMA-style protocol. Studies were considered eligible for inclusion if they reported empirical, survey-based, or qualitative data on factors influencing specialty or career choice, career selection, or workforce recruitment in ophthalmology or surgery more broadly, in undergraduate or postgraduate populations; peer-reviewed journal articles, professional body workforce reports, and relevant gray literature (e.g. national recruitment statistics) were all eligible for inclusion, with no restriction placed on study design. Studies were excluded if they addressed specialty choice in non-surgical, non-ophthalmic disciplines without clear relevance to the review's themes, were not available in English, or consisted of opinion pieces or editorials without underlying primary data.

The search of PubMed/MEDLINE, Embase, and Google Scholar, supplemented by manual screening of reference lists and relevant literature, initially identified several potentially relevant records. Titles and abstracts were screened by the author for thematic relevance to career choice and workforce planning in ophthalmology and surgery; the full texts of candidate articles were then reviewed in detail, and a final set of 28 sources judged most directly relevant and of adequate methodological quality was retained for synthesis, spanning both general surgical career choice literature and ophthalmology-specific studies. This selection process was undertaken by a single author and consistent with the narrative rather than systematic format of this review.

Because no formal critical appraisal instrument (such as GRADE or the Mixed Methods Appraisal Tool) was applied, the quality of the included evidence was assessed informally against criteria, including sample size, response rate, study design, source credibility (peer-reviewed publication versus gray literature), and the consistency of findings across independent studies and settings. Ophthalmology-specific evidence was prioritized over extrapolation from the general surgical literature wherever it was available, and findings from single, small, or methodologically limited studies were weighted more cautiously than those from well-powered surveys or results replicated across multiple independent studies. Readers should therefore interpret the strength of evidence underlying individual claims in this review in light of this pragmatic, author-judgment-based approach to quality assessment, rather than as the product of a formal, reproducible systematic review methodology.

## Review

The influence of role models and mentors

Across surgical specialties, the presence of enthusiastic, accessible role models at the undergraduate level emerges as one of the most consistently reported drivers of specialty interest. A 2012 study of general surgery residents, resident applicants, and medical students confirmed that clerkship role models and mentors directly shaped interest in surgical training, with resident applicants holding this view even more strongly than undecided students [7]. The mechanism appears to be dual: professional identification -- whereby students visualize themselves in the role of the operating surgeon -- and practical career guidance regarding training pathways, examinations, and long-term prospects.

In ophthalmology, this dynamic carries particular force. Because ophthalmology rotations are often severely compressed or entirely absent from undergraduate medical curricula, the quality of contact students do have with ophthalmologists exerts disproportionate influence on career intentions. A single memorable encounter -- being invited to examine fundal pathology at the slit lamp, observing a vitreoretinal procedure, or hearing a consultant articulate the intellectual fascination of neuro-ophthalmology -- can be pivotal. Conversely, dismissive or unwelcoming faculty behavior has been identified as a significant deterrent across surgical specialties and must be actively guarded against [8].

Surgeons, including ophthalmologists, have been demonstrated to systematically underestimate their own influence on students who are considering a surgical career [7]. Faculty development programs that sensitize consultants to their mentoring role, and that formally reward excellence in undergraduate teaching, are therefore likely to yield substantial dividends for recruitment. Structured one-to-one mentorship schemes -- connecting medical students with ophthalmology specialty trainees and consultants -- provide a scalable means of sustaining the interest generated by positive early encounters. Virtual mentoring platforms have extended the reach of such programs beyond geographic constraints, enabling students in institutions without ophthalmology departments to access specialist guidance.

The distinction between a role model and a mentor is worth noting in this context. A role model inspires through their professional identity and demeanor; a mentor actively invests in a student's development through advice, advocacy, and sponsorship. Ophthalmology benefits from promoting both: charismatic role models who communicate the rewards of the specialty, and dedicated mentors who help students navigate the practical steps toward a career in it.

Undergraduate exposure and medical school placements

The extent and quality of clinical exposure during medical school is among the strongest independent predictors of subsequent career choice in surgery. An observational study of over 100 medical students found that the number of hours spent in the operating theater was significantly correlated with the decision to pursue a surgical career [9]. The quality of attending teaching, the opportunity to participate meaningfully in operative procedures, and the cultivation of a supportive learning environment further amplify this effect; and positive mentor-mentee relationships formed during clerkship are associated with sustained specialty interest [5].

Ophthalmology faces a structural challenge in this regard. In many medical school curricula, the ophthalmology placement has been compressed to a matter of days -- or eliminated altogether in favor of community-based primary care attachments -- as part of broader reform processes emphasizing generalist competencies [10]. This contraction has occurred at precisely the moment when the specialty has become more technically sophisticated, more evidence-based, and more capable of transforming patient outcomes. Students who graduate without having examined a fundus, witnessed a cataract list, or encountered a patient whose independence has been restored by timely retinal reattachment surgery are unlikely to develop the informed enthusiasm necessary to consider ophthalmology as a career destination [11-14].

Training structure and the assurance of adequate case exposure are documented concerns for trainees deciding on a surgical specialty [8]. For ophthalmology specifically, this translates into the importance of wet-laboratory microsurgery courses, simulation-based surgical training, and transparent competency frameworks that allow trainees to track their progression. Faculty diversity -- with representation across subspecialties, including pediatric ophthalmology, medical retina, glaucoma, cornea, oculoplastics, and neuro-ophthalmology -- broadens the appeal of the specialty and signals the range of career trajectories available to trainees [15].

Evidence from general surgery suggests that non-metropolitan or district general hospital placements, though often perceived as less prestigious than major teaching center attachments, provide richer mentorship, a more supportive learning environment, and greater operative experience per student [16]. A comparable purposefully designed ophthalmology placement in district settings could expand the pool of students who consider the specialty, particularly those from non-urban or less privileged backgrounds who may not have access to leading ophthalmology centers.

Stability of career preference and competition from other specialties

Career preference among medical students is not immutable. Longitudinal data indicate that approximately 60% of students maintain their initial specialty preference through the course of their medical training, while the remainder shift -- often in response to new clinical experiences, changing personal circumstances, or revised perceptions of lifestyle and income [15,17]. For ophthalmology, this underscores the value of engaging prospective trainees at multiple timepoints throughout their undergraduate and early postgraduate years, rather than relying on a single formative experience.

Ophthalmology competes for trainees with other specialties perceived as offering a favorable combination of intellectual stimulation, procedural variety, lifestyle, and financial reward. United States data from the 1996-2002 period documented rising interest in dermatology, radiology, and anesthesia among medical graduates at the expense of general surgery [15]. Ophthalmology -- not a direct comparator in those analyses -- nonetheless operates within the same competitive landscape. The specialty possesses a distinctive profile that should be actively communicated: procedures are microsurgical and deeply satisfying, outcomes are frequently immediate and dramatic, and the predominance of elective outpatient and theater work affords a degree of schedule predictability that many trainees value.

Naylor et al. identified a worrying longitudinal decline in positive perceptions of surgeons as students progressed from third to fourth year of medical school, suggesting that enthusiasm generated during a single clerkship may attenuate without deliberate maintenance [18]. Ophthalmology training programs and specialty societies can counteract this trajectory by sustaining contact with interested students through funded research intercalated degrees, taster weeks, visiting speaker programs, and invitations to attend regional and national conferences. Daly et al. similarly recommend prolonging positive surgical experiences through mentorship, operative exposure, and engagement with research [5].

The role of student ophthalmology societies

Student surgical and specialty societies have been shown to exert a measurable positive influence on career intentions. A study conducted at three UK medical schools demonstrated that membership of a student surgical society increased awareness of surgical subspecialties and left a lasting positive impression on career planning, partially compensating for the recession of anatomy and surgical teaching from undergraduate curricula [19]. Career-guiding events, practical skills workshops, and opportunities to hear from trainees across different career stages were identified as particularly effective elements of society activity.

Student ophthalmology interest groups -- operating under the auspices of national bodies such as the Royal College of Ophthalmologists (RCOphth) in the United Kingdom or the American Academy of Ophthalmology (AAO) -- provide a valuable platform for early engagement. Practical activities such as basic slit-lamp training sessions, wet-laboratory days using model eyes and Perspex blocks, retinal photography interpretation workshops, and site visits to low-vision rehabilitation services broaden students' understanding of ophthalmology beyond the operating theater. Such activities may attract students whose primary motivation is the prevention of blindness as a global health challenge rather than surgical craft per se [20].

Sutton et al., in a national UK study examining the attitudes, motivators, and barriers to surgical careers among undergraduate medical students, identified five themes central to improving careers support: increased exposure during clinical attachments, dedicated careers lectures, careers fairs, earlier access to careers information, and structured mentorship [21]. Each of these themes translates directly into actionable activity for ophthalmology societies and the specialty's professional bodies.

It is important to separate three distinct issues that are often conflated under the umbrella of "workforce concerns": student interest in the specialty, the competitiveness of specialty-training applications, and workforce supply. These do not necessarily move together, and in some settings, they point in opposite directions. In the United Kingdom, for example, official 2025 national recruitment data list Ophthalmology ST1 as receiving 2,197 applications for 102 posts, a competition ratio of 21.54 -- among the highest of any UK specialty training program and one that has risen sharply in recent recruitment cycles. This indicates that, at least in the UK context, ophthalmology suffers not from a shortage of interested applicants but from intense competitiveness at the point of specialty selection. A genuine workforce shortfall in such settings is therefore unlikely to reflect low student interest; it more plausibly relates to constraints on training capacity (the number of funded training posts), retention of trained ophthalmologists within the workforce, the geographic distribution of consultants and services, and rising service demand driven by an aging population. Societies and educators should accordingly be cautious about framing all recruitment activity as a response to "declining interest," since in highly competitive settings, the more pressing task may be expanding training capacity and improving retention and distribution rather than generating further undergraduate enthusiasm for a specialty that already attracts many more applicants than it can accommodate.

Lifestyle, work-life balance, and the demands of ophthalmic training

Lifestyle considerations are among the most powerful determinants of specialty choice across all medical disciplines. Research has consistently demonstrated that concerns about work-life balance, long working hours, and impact on family and personal life are major deterrents to surgical careers. Wendel et al. found that practice lifestyle deterred 83% of male and 63% of female medical students from pursuing surgery [22]. A survey of surgical program directors found that over half of respondents' programs were affected by trainee attrition, with lifestyle-related problems accounting for the majority of departures [15]. Poor work-family balance has been linked to higher rates of burnout, increased prevalence of depression, and reduced career satisfaction -- outcomes that impose costs both on the individuals concerned and on the healthcare systems that trained them [8].

Ophthalmology occupies a comparatively favorable position within the surgical landscape in this respect. Emergency out-of-hours work, including the management of acute angle-closure glaucoma, open-globe trauma, endophthalmitis, and retinal detachment, is present but less all-consuming than the on-call burden of general, vascular, or trauma surgery. The predominance of elective outpatient clinic work and planned surgical lists confers a degree of schedule predictability that many trainees value highly, and that may be underappreciated by medical students who conflate all surgical specialties into a single high-demand category. This comparative advantage should be clearly and proactively communicated during undergraduate education and careers events.

Nevertheless, the cumulative demands of ophthalmology training remain significant. The acquisition of complex microsurgical skills requires sustained practice beyond formal working hours. The competitiveness of specialty selection -- with high achievement thresholds at the foundation and core training levels -- generates substantial pressure for junior doctors. Research productivity is increasingly expected of applicants. Structural interventions, including flexible training pathways, formally recognized part-time training options, and clearly defined progression frameworks, are important for retaining trainees who might otherwise leave the specialty due to competing personal demands.

Perceived future income, social prestige, and career opportunities

Financial considerations and social prestige constitute a complex and evolving set of motivators in specialty selection. Research has repeatedly shown that the income differential between surgery and less-demanding specialties is narrowing in many healthcare systems, eroding a traditional incentive for surgical careers [15]. In the United States, the debt burden carried by medical graduates has grown substantially over the decades since Azizzadeh et al. documented graduate debt figures of $80,000 and $110,000 from public and private institutions, respectively, in 1999 [1] and now frequently exceeds six figures. The financial calculus of choosing a competitive specialty with a longer training pathway, against the relative security of general practice or a less demanding hospital specialty, may weigh against ophthalmology for some debt-averse graduates.

Ophthalmology nonetheless retains considerable social prestige, grounded in its technical complexity, the clarity of its patient outcomes, and the high value placed by society on the preservation and restoration of sight. Career diversification within the specialty further enhances its appeal: subspecialty pathways span academic and clinical medicine, industry collaboration in ophthalmic device and pharmaceutical development, global eye health program leadership, private practice, and medicolegal work, offering a breadth of trajectory that resonates with students of varying professional ambitions.

Career prestige and varied professional opportunities -- including access to research, teaching, and leadership roles -- are recognized as positive attractors to surgical careers and should be actively promoted through career materials and events [8]. For ophthalmology, the narrative of a specialty at the frontier of medical science -- from retinal gene therapy to AI-enabled screening -- is a compelling one that medical educators and specialty societies should communicate with confidence.

Intrinsic motivation: the "calling" to ophthalmology

Beyond the extrinsic determinants of income, lifestyle, and prestige, a profound personal fit with the specialty -- a "calling" -- consistently emerges in the literature as the single most important positive driver of surgical career choice. A survey of Swiss board-certified surgeons identified fascination with the field, the use of manual and varied skills, meaningful patient care, and the application of technology as the most compelling reasons for choosing surgery [8,23]. Teamwork, the intellectual challenge of complex cases, the meaningfulness and responsibility of the role, and the dynamic and evolving nature of the discipline were additionally cited as positive factors that sustained long-term career satisfaction.

In ophthalmology, these intrinsic rewards are unusually vivid. The restoration of functional vision through cataract surgery delivers immediate, tangible, and deeply gratifying feedback that few other medical interventions can rival. The patient who removes their dressing the morning after surgery and sees clearly for the first time in years represents one of medicine’s most powerful narratives. Equally compelling is the intellectual richness of a specialty that sits at the interface between medicine and surgery, between molecular biology and public health: the ophthalmologist who identifies hypertensive retinopathy, manages a patient with ocular manifestations of multiple sclerosis, or contributes to a clinical trial of a novel anti-vascular endothelial growth factor agent is engaged in the practice of genuine breadth [6,24].

Whilst the most commonly cited discouraging factors in surgical careers broadly are excessive workload (cited by 37.7%), training difficulties (31.3%), and inability to maintain work-life balance (14.5%), the intrinsic calling to the specialty remains the key positive factor that ultimately overrides these obstacles for those who commit to it [8,23]. This finding has an important practical implication: efforts to inspire genuine passion for ophthalmology -- through compelling clinical encounters, patient-centered narratives, and engagement with the specialty's research agenda -- may be as powerful as, or more powerful than, structural reforms to the training pathway in attracting the next generation of ophthalmologists.

Cost, financial burden, and barriers to training

The financial burden associated with specialist surgical training is substantial across disciplines. Mandatory training courses, postgraduate examination fees, professional membership subscriptions, and conference attendance impose costs on trainees during the relatively lower-income years of postgraduate training [1]. For ophthalmology specifically, the acquisition of microsurgical skills often requires access to wet-laboratory facilities and simulation equipment, and attendance at phacoemulsification training courses and other mandatory practical assessments, none of which are uniformly funded by deaneries or training programs.

 The functional support available from training schools to assist trainees in completing mandatory requirements has been criticized as inadequate in some settings, and this concern is relevant to ophthalmology as much as to other surgical disciplines [1]. Transparency about the true financial cost of an ophthalmology training pathway -- and the provision of bursaries, funded courses, and competitive salary structures that recognize the investment trainees make -- is an important component of equitable recruitment. Without such transparency, the true cost of a surgical career may deter talented candidates from less financially secure backgrounds, exacerbating socioeconomic inequities in the consultant workforce.

Medical school tuition fees and associated graduate debt create a backdrop against which every subsequent career decision is made. Students carrying substantial educational debt may rationally prioritize income security and a shorter pathway to consultant-level earnings over the intellectual and procedural rewards of ophthalmology training. Scholarship programs, sponsored research degrees, and advocacy for trainee pay parity within ophthalmology are partial but meaningful responses to this challenge.

Gender, diversity, and inclusion in ophthalmic career choice

The increasing proportion of women entering medical school over the past three decades has substantially transformed the demographic composition of the medical workforce [15]. Ophthalmology has, in many countries, made notable progress toward gender balance among trainees and early career consultants. Positive clerkship experiences, the visibility of female role models across consultant and leadership grades, enthusiastic and equitable teaching, and active involvement in the operating theater have been identified as key factors in attracting women to surgical careers [15]. The presence of female mentors, in particular, is associated with greater confidence and clearer professional identity among female students considering surgical specialties.

Nevertheless, persistent disparities remain. Women may be deterred from ophthalmology by perceptions -- sometimes well-founded - of a masculine professional culture, a relative paucity of senior female role models in certain subspecialties, and concerns about balancing the demands of training with family responsibilities and maternity leave [22,25]. Research across surgical specialties has consistently documented differential treatment of male and female trainees in the allocation of operative cases, feedback practices, and access to career sponsorship. Such differential treatment undermines performance, damages morale, contributes to attrition, and discourages talented female students from entering the specialty [15,26-28]. Ophthalmology must commit to zero-tolerance policies for discriminatory behavior and active monitoring of training equity.

Initiatives analogous to the Royal College of Surgeons of England's Women in Surgery (WiNS) program or the Perry Initiative in the United States, tailored specifically to ophthalmology and sustained with adequate resourcing, represent important systemic interventions. Diversity and inclusion in ophthalmology must, however, extend beyond gender to encompass race and ethnicity, socioeconomic background, disability status, and other dimensions of identity that shape access to the role models, networks, and experiences that determine specialty choice. A genuinely inclusive recruitment approach -- one that actively identifies and dismantles structural barriers rather than assuming a level playing field -- is both an ethical imperative and a strategic necessity for a specialty that aspires to reflect and serve a diverse patient population (Table 1).

**Table 1:** Summary of the major motivating factors and barriers

Domain	Major motivating factors	Major barriers
Undergraduate exposure	Meaningful clinical placements; hands-on procedures (slit-lamp examination, theater attendance, wet-lab microsurgery)	Compressed or absent ophthalmology placements in undergraduate curricula
Role models and mentorship	Enthusiastic, accessible role models; structured one-to-one and virtual mentorship schemes	Insufficient structured mentorship; faculty underestimation of their own influence; dismissive faculty behavior
Lifestyle and work-life balance	Comparatively predictable, elective-dominated schedule relative to other surgical specialties	Sustained out-of-hours practice required for microsurgical skill acquisition; competing personal demands during training
Finances, prestige, and career opportunities	High social prestige; diverse career trajectories (academia, industry, global health, private practice)	Training course, examination and conference costs; rising graduate debt burden
Specialty selection and competition	Strong sustained applicant interest, reflecting the specialty's desirability	High competition ratios at specialty selection; limited funded training posts
Gender, diversity, and inclusion	Growing female representation among trainees; visible female role models and leaders	Perceived masculine professional culture; differential treatment; socioeconomic and other structural inequities
Intrinsic motivation	A personal "calling"; immediate, tangible patient outcomes (e.g., cataract surgery)	Calling may never develop without early, meaningful exposure to the specialty

Discussion

The global trend of declining interest in surgical careers, extensively documented in the general surgery literature, provides a cautionary template for ophthalmology. The specialty possesses inherent strengths as a career destination: intellectual richness, technical demand, rapid evolution, meaningful patient outcomes, and a comparatively attractive lifestyle profile within surgery. Yet without deliberate and sustained effort to communicate these advantages and to remove structural barriers to entry, the ophthalmic workforce risks progressive inadequacy in the face of rising global visual impairment.

Based on this synthesis of the literature, several broad evidence-informed recommendations emerge. Early and meaningful clinical exposure, including slit-lamp examination, operating theater attendance, and simulation-based microsurgery, should be embedded in undergraduate medical curricula rather than treated as optional enrichment. Structured, inclusive mentorship programs connecting students with ophthalmology trainees and consultants at all career stages can sustain interest beyond a single formative clerkship. Student ophthalmology societies, actively supported by royal colleges and professional associations, can bridge the gap created by compressed undergraduate curricula and provide the career guidance, practical skills, and networking opportunities that help students make informed choices.

Addressing gender and diversity barriers demands active and visible sponsorship of underrepresented trainees, zero tolerance for discriminatory behavior in training environments, and flexible training pathways that do not penalize trainees with caring responsibilities. Transparent communication about the lifestyle profile of ophthalmology -- and about the remarkable breadth of career trajectories available within the specialty -- will help prospective trainees make informed decisions. The financial burden of ophthalmic training should be acknowledged and, where possible, mitigated through funded courses, subsidized examination preparation, and equitable trainee remuneration.

The “Shape of Training” report in the United Kingdom and comparable reform agendas in other health systems, which emphasize generalist competencies at the expense of specialist exposure, pose an ongoing structural challenge for recruitment to procedurally intensive specialties such as ophthalmology. The specialty’s advocates -- trainees, consultants, royal colleges, patient groups representing those living with sight loss, and healthcare commissioners -- must engage proactively with these policy debates to safeguard the specialty’s place in the undergraduate curriculum and to ensure that the next generation of ophthalmologists is both numerically sufficient and clinically equipped to meet the demands of the twenty-first century.

## Conclusions

The forces shaping career choice in ophthalmology are both multifactorial and modifiable. The motivating factors -- intrinsic passion for the field, inspiring role models, meaningful operative exposure, rewarding patient outcomes, and an attractive lifestyle profile relative to other surgical disciplines -- are real and powerful, but they require deliberate cultivation and active promotion. Equally, the barriers identified in this review -- limited undergraduate exposure, financial burden, competitive selection, gender and socioeconomic inequities, and insufficient mentorship -- are not immutable features of the specialty but addressable failures of system design and institutional culture.

A coordinated response from medical schools, postgraduate training bodies, royal colleges, and ophthalmology’s professional community is required. Investment in early exposure, structured mentorship, inclusive recruitment practices, and transparent career information is not merely good practice -- it is a strategic necessity for a specialty that aspires to remain vibrant, equitable, and equal to the challenges of twenty-first-century eye care.
